# HEIGHTENED STRESS IN FOOD CHOICES: HOW ACUTE EXERCISE IMPACTS OLDER ADULTS’ DECISION-MAKING

**DOI:** 10.1093/geroni/igae098.4203

**Published:** 2024-12-31

**Authors:** Hyeon Jung (Judith) Heselton, Annelie Persson, Julie Blaskewicz Boron

**Affiliations:** University of Nebraska Omaha, Lincoln, Nebraska, United States; University of Nebraska, Omaha, Omaha, Nebraska, United States; University of Nebraska Omaha, Omaha, Nebraska, United States

## Abstract

Both habitual and acute exercise have a subjective stress-reducing effect in young and older adults. Exercise is associated with improved cognitive performance related to decision-making, while stress correlates with poorer decision-making regarding food intake in some individuals. Previous research with young adults found that acute exercise reduced perceived stress, as well as increased the amount selected for consumption both immediately after and 30 minutes post exercise. The current study assessed the impact of acute exercise on perceived stress in older adults while making hypothetical choices related to the amount and timing of food intake. Thirty-one healthy older (Mage=68.67; SD=3.08; Range=64-75) participants (54.8% female; Mean BMI=26.16kg/m2; SD=2.82kg/m2) completed 45-minutes of aerobic exercise and a resting control condition in randomized order. Perceived stress was surveyed (scale 0-10) before the exercise bout, immediately after, and 30-minutes post-condition. Food amount and intertemporal food preferences (e.g., immediate vs. delayed consumption) were assessed. Repeated-measures of analysis of variance (SPSS version 29) indicated no main effect on food amount preference, preferred timing of consumption, or stress; however, an interaction of condition and time on stress (p=0.013) showed when compared to rest, exercise resulted in increased stress from pre-to-post+30 min (F=7.58; p=0.02). Results suggest that acute exercise may transiently increase perceived stress in the context of decision-making around food choices in older adults highlighting the importance of available food options for this population. After replication, future studies should seek to elucidate the etiology and duration of increased stress, and the source of stress, in older adults.

